# Supplementary material for: A Meta-Analysis of the Converging Effects of Different Classes of Antipsychotics on the Frontal Cortex Transcriptome in Laboratory Rodents and Non-Human Primates
Source: bioRxiv. 2026 Jun 29:2026.06.24.734301. Preprint. [Version 1] doi: 10.64898/2026.06.24.734301 (PMC13345317; doi:10.64898/2026.06.24.734301)
Supplement: Supplement 1 [file media-1.pdf]

<u>Supplemental Results for:</u>

A Meta-Analysis of the Converging Effects of Different Classes of Antipsychotics on the Frontal Cortex Transcriptome in Laboratory Rodents and Non-Human Primates

Mubashshir Ra'eed Bhuiyan[1], Megan Hastings Hagenauer[1], Eva Geoghegan[2], Elizabeth Flandreau[3], Stanley Watson Jr.[1], Huda Akil[1]

1. University of Michigan, Ann Arbor, MI 48109 USA
2. University of Cambridge, Cambridge, United Kingdom
3. Grand Valley State University, Allendale, MI 49401 USA

Supplemental Table Legends

Table S1. The input used for the meta-analyses. *This .xlsx file includes 3 worksheets: 1) The worksheet "Column Definitions" contains the definitions for each of the statistical worksheets and columns. 2) The worksheet "Log2FCs" provides the average log(2) fold changes for each gene for each of the antipsychotic treatment vs. control comparisons included in either the planned rodent meta-analysis or exploratory rodent-macque meta-analysis. In order to be included within either the planned rodent meta-analysis or exploratory rodent-macaque meta-analysis, a gene needed to be present within at least 7 of the available antipsychotic treatment vs. control comparisons. The gene annotation for each of the 13,121 rows is provided for each relevant species (mouse, rat, macaque (with human)), as well as information about the evidence supporting gene orthology across species. The antipsychotic treatment vs. control comparisons are identified by a dataset identifier (either gene expression omnibus accession# or research consortium) and treatment name. The Log2FCs were averaged if there were multiple results available for any particular gene (as identified by mouse Entrez ID, rat Entrez ID, or the mouse Entrez ID for the respective macaque/human ortholog). 3) The worksheet "SamplingVariances" provides the sampling variances for each gene to accompany the Log2FCs in worksheet #1 (13,121 genes). The sampling variances were calculated as the square of the standard error (Log2FC/T-statistic). The standard errors were averaged if there were multiple results available for any particular gene (as identified by mouse Entrez ID, rat Entrez ID, or the mouse Entrez ID for the respective macaque/human ortholog).*

Table S2. Planned meta-analysis results: The full results from the planned meta-analysis of antipsychotic effects on the frontal cortex of rodents (12,215 genes, 12,190 stable meta-analysis estimates). *This .xlsx file includes two worksheets: 1) The worksheet "ColumnDefinitions" provides the definitions for the variables present in each column in "PlannedRodentMetaAnalysisOutput". 1) The worksheet "PlannedRodentMetaAnalysisOutput" provides the full meta-analysis results, with each row representing the results for one gene, and each column providing either gene annotation or meta-analysis statistical output. The results are ordered by p-value, so that the top rows in the worksheet are the genes with the smallest p-values.*

Table S3. Planned meta-analysis results: All 45 genes consistently upregulated in the frontal cortex following antipsychotic treatment in rodents (FDR<0.05). *Results are ordered by estimated Log2FC (Log2 Fold Change of Antipsychotic treatment vs. Control); a higher Log2FC indicates upregulation of the gene within the frontal cortex following antipsychotic treatment compared to the control group. Given gene symbols are the mouse symbol for the respective gene. Definitions: Log2FC = Log2 Fold Change, SE = Standard Error, CI_lb=confidence interval lower bound, CI_ub=confidence interval upper bound, p-value = nominal p-value, FDR = False Discovery Rate (q-value). The #Comparisons represents the number of treatments that included measurements for the gene following quality control. The "Confirmed?" column indicates whether a gene continued to show differential expression in the larger exploratory meta-analysis drawing from data from both rodents and macaques - a single asterisk (*) indicates that the gene continued to show significant differential expression (FDR<0.05) in the larger exploratory meta-analysis, regardless of whether macaque data was available, whereas double asterisks (**) indicate that the gene was both significant and had rhesus macaque data available. Full results for all genes can be found in* ***Table S2****.*

Gene Symbol	Mouse Entrez Gene ID	Log2FC estimate	SE	pval	CI_lb	CI_ub	#Comparisons	FDR	Confirmed?
Nkx6-2	14912	0.288	0.0608	2.06E-06	0.169	0.408	8	5.03E-03	**
Plekhh1	211945	0.274	0.0697	8.40E-05	0.137	0.410	8	2.44E-02	
Gjb1	14618	0.271	0.0588	4.04E-06	0.156	0.386	8	7.04E-03	*
Mfap2	17150	0.246	0.0390	2.99E-10	0.169	0.322	8	1.83E-06	**
Igfbp5	16011	0.238	0.0564	2.47E-05	0.127	0.348	8	1.40E-02	**
Mdc1	240087	0.228	0.0508	7.59E-06	0.128	0.327	8	7.27E-03	
Cdc42ep2	104252	0.210	0.0470	8.33E-06	0.117	0.302	8	7.27E-03	
Prok1	246691	0.205	0.0519	7.66E-05	0.104	0.307	7	2.34E-02	*
Ccdc71	72454	0.200	0.0384	1.87E-07	0.125	0.276	7	5.70E-04	**
Gh	14599	0.192	0.0496	1.11E-04	0.095	0.289	8	2.87E-02	*
Osgin1	71839	0.186	0.0492	1.59E-04	0.089	0.282	8	3.60E-02	**
Pola2	18969	0.186	0.0408	5.49E-06	0.106	0.266	8	7.27E-03	
Cpne7	102278	0.185	0.0436	2.25E-05	0.099	0.271	8	1.40E-02	
Rlbp1	19771	0.183	0.0411	8.13E-06	0.103	0.264	8	7.27E-03	
Aspa	11484	0.182	0.0432	2.41E-05	0.098	0.267	8	1.40E-02	**
Slc18a3	20508	0.182	0.0457	6.85E-05	0.092	0.272	8	2.34E-02	*
Tdo2	56720	0.181	0.0404	7.78E-06	0.102	0.260	8	7.27E-03	*
1700010I14Rik	66931	0.172	0.0412	3.10E-05	0.091	0.253	8	1.40E-02	*
Gpr84	80910	0.171	0.0417	4.27E-05	0.089	0.253	8	1.80E-02	*
Slc14a1	108052	0.170	0.0376	6.27E-06	0.096	0.243	8	7.27E-03	**
Slc1a5	20514	0.159	0.0371	1.95E-05	0.086	0.231	8	1.40E-02	*
Tppp3	67971	0.159	0.0400	7.28E-05	0.080	0.237	8	2.34E-02	
Tspo	12257	0.159	0.0378	2.70E-05	0.085	0.233	8	1.40E-02	
Slc9a8	77031	0.155	0.0392	7.14E-05	0.079	0.232	7	2.34E-02	
Plekhh3	217198	0.148	0.0386	1.23E-04	0.073	0.224	8	3.01E-02	
Sgcg	24053	0.148	0.0384	1.23E-04	0.072	0.223	7	3.01E-02	
F10	14058	0.145	0.0382	1.45E-04	0.070	0.220	7	3.47E-02	**
Cers2	76893	0.145	0.0362	6.42E-05	0.074	0.215	8	2.31E-02	
Cmtm3	68119	0.144	0.0360	5.89E-05	0.074	0.215	8	2.25E-02	**
Mtmr14	97287	0.142	0.0302	2.78E-06	0.082	0.201	8	5.67E-03	
Gtse1	29870	0.142	0.0366	1.08E-04	0.070	0.213	8	2.87E-02	*
Slc22a5	20520	0.141	0.0337	3.04E-05	0.075	0.207	8	1.40E-02	**
Mrgprf	211577	0.140	0.0353	6.93E-05	0.071	0.209	8	2.34E-02	**
Plekhj1	78670	0.139	0.0355	8.91E-05	0.070	0.209	8	2.47E-02	
Actl6b	83766	0.127	0.0302	2.62E-05	0.068	0.186	8	1.40E-02	
Rbp7	63954	0.123	0.0329	1.82E-04	0.059	0.187	7	3.96E-02	*
Tmem134	66990	0.118	0.0278	2.38E-05	0.063	0.172	8	1.40E-02	**
Tbc1d17	233204	0.117	0.0264	9.77E-06	0.065	0.168	8	7.96E-03	
Fam234a	106581	0.116	0.0306	1.56E-04	0.056	0.176	8	3.60E-02	*
Cd180	17079	0.110	0.0280	8.36E-05	0.055	0.165	8	2.44E-02	**
Hnrnpl	15388	0.109	0.0291	1.69E-04	0.052	0.166	8	3.76E-02	*
Pcyt2	68671	0.100	0.0269	1.85E-04	0.048	0.153	8	3.96E-02	
Kctd2	70382	0.100	0.0237	2.42E-05	0.054	0.146	7	1.40E-02	
Polr2c	20021	0.100	0.0144	4.50E-12	0.071	0.128	8	5.49E-08	
Txndc15	69672	0.069	0.0182	1.56E-04	0.033	0.104	8	3.60E-02	

Table S4. Planned meta-analysis results: All 18 genes consistently downregulated in the frontal cortex following antipsychotic treatment in rodents (FDR<0.05). *Table follows the conventions of* ***Table S3****. Results are ordered by estimated Log2FC (Log2 Fold Change of Antipsychotic treatment vs. Control); a lower Log2FC indicates downregulation of the gene within the frontal cortex following antipsychotic treatment compared to the control group. Full results for all genes can be found in* ***Table S2****.*

Gene Symbol	Mouse Entrez Gene ID	Log2FC estimate	SE	pval	CI_lb	CI_ub	#Comparisons	FDR	Confirmed?
Ms4a8a	64381	-0.248	0.0427	6.28E-09	-0.331	-0.164	7	2.56E-05	*
Plcg2	234779	-0.230	0.0573	5.86E-05	-0.343	-0.118	8	2.25E-02	
Sowaha	237761	-0.203	0.0484	2.89E-05	-0.298	-0.108	7	1.40E-02	
Atp8a2	50769	-0.192	0.0499	1.20E-04	-0.290	-0.094	7	3.01E-02	
Gpr45	93690	-0.179	0.0482	2.08E-04	-0.273	-0.084	8	4.17E-02	
Fabp4	11770	-0.168	0.0417	5.36E-05	-0.250	-0.087	7	2.18E-02	**
Ica1l	70375	-0.160	0.0435	2.34E-04	-0.245	-0.075	7	4.60E-02	
Tnfsf10	22035	-0.148	0.0403	2.47E-04	-0.227	-0.069	7	4.79E-02	
Ar	11835	-0.137	0.0369	2.02E-04	-0.210	-0.065	8	4.17E-02	**
Tasp1	75812	-0.136	0.0347	8.65E-05	-0.204	-0.068	8	2.46E-02	**
Nt5c	50773	-0.136	0.0349	1.01E-04	-0.204	-0.067	8	2.73E-02	
Nol4l	329540	-0.123	0.0307	6.29E-05	-0.183	-0.063	7	2.31E-02	
Ncr1	17086	-0.120	0.0290	3.44E-05	-0.177	-0.063	7	1.50E-02	*
Sorbs2	234214	-0.118	0.0260	5.94E-06	-0.169	-0.067	8	7.27E-03	
Nudt7	67528	-0.117	0.0315	2.06E-04	-0.179	-0.055	8	4.17E-02	**
Cdk5r1	12569	-0.104	0.0280	2.08E-04	-0.159	-0.049	8	4.17E-02	
B4galnt1	14421	-0.104	0.0262	7.64E-05	-0.155	-0.052	8	2.34E-02	
Pprc1	226169	-0.089	0.0213	2.92E-05	-0.131	-0.047	8	1.40E-02	

Table S5. Exploratory results from a meta-regression including antipsychotic type (second-generation vs. first-generation) as a co-variate (12,215 genes, 12,203 stable meta-analysis estimates). *This .xlsx file includes two worksheets: 1) The worksheet "ColumnDefinitions" provides the definitions for the variables present in each column in "ExploratoryMetaRegressionResults". 1) The worksheet "ExploratoryMetaRegressionResults" provides the full meta-analysis results, with each row representing the results for one gene, and each column providing either gene annotation or meta-analysis statistical output. The results are ordered by p-value for the overall effect of the covariate antipsychotic type, so that the top rows in the worksheet are the genes with the smallest p-values.*

Table S6. 39 genes showed significant evidence of publication bias when considering the full meta-analysis output (Egger's test FDR<0.05). *The Egger's regression test is used to detect funnel plot asymmetry, which is a commonly used indicator of publication bias. Rows in the table are ordered by the p-value from the Egger's regression test ("Egger Pval"), provided with the associated Z statistic ("Egger Zstat") and false discovery rate ("Egger FDR"). Other columns follow the conventions used in* ***Table S3*** *and* ***Table S4****, but with "AP" listed before each statistic to signify "Antipsychotic Effect". Full results for all genes can be found in* ***Table S2****.*

Gene Symbol	Mouse Entrez Gene ID	AP Log2FC estimate	AP SE	AP pval	AP CI_lb	AP CI_ub	# Comparisons	AP FDR	Egger Zstat	Egger Pval	Egger FDR
Tepsin	78777	0.28	0.32	3.69E-01	-0.34	0.90	8	8.26E-01	5.30	1.15E-07	1.24E-03
Strip2	320609	-0.04	0.12	7.39E-01	-0.28	0.20	7	9.51E-01	-5.13	2.87E-07	1.24E-03
Utp14a	72554	-0.08	0.06	1.93E-01	-0.20	0.04	8	7.01E-01	-5.12	3.04E-07	1.24E-03
Tbx18	76365	0.00	0.12	9.91E-01	-0.24	0.24	8	1.00E+00	4.96	6.94E-07	2.12E-03
Polm	54125	0.02	0.07	7.13E-01	-0.11	0.15	8	9.48E-01	4.75	1.99E-06	4.86E-03
Tradd	71609	-0.26	0.14	6.54E-02	-0.54	0.02	8	5.06E-01	-4.65	3.35E-06	6.82E-03
Nab2	17937	-0.01	0.07	8.78E-01	-0.15	0.13	8	9.83E-01	4.58	4.72E-06	8.23E-03
Dph2	67728	0.01	0.06	9.13E-01	-0.11	0.12	8	9.87E-01	-4.47	7.76E-06	1.19E-02
Wsb1	78889	0.15	0.12	2.00E-01	-0.08	0.39	8	7.09E-01	4.38	1.18E-05	1.60E-02
Tiparp	99929	-0.09	0.12	4.69E-01	-0.33	0.15	8	8.70E-01	4.32	1.55E-05	1.69E-02
Fancd2os	70979	0.04	0.10	6.68E-01	-0.16	0.24	7	9.35E-01	-4.31	1.67E-05	1.69E-02
Unc5a	107448	-0.03	0.08	7.05E-01	-0.18	0.12	8	9.45E-01	4.30	1.72E-05	1.69E-02
Slc7a2	11988	0.01	0.06	8.59E-01	-0.11	0.13	8	9.78E-01	-4.28	1.84E-05	1.69E-02
Folr1	14275	-0.37	0.23	1.03E-01	-0.81	0.08	8	5.79E-01	-4.27	1.97E-05	1.69E-02
Prl4a1	19110	-0.35	0.25	1.71E-01	-0.84	0.15	7	6.78E-01	-4.23	2.32E-05	1.69E-02
Sympk	68188	0.08	0.06	1.47E-01	-0.03	0.19	7	6.48E-01	4.23	2.32E-05	1.69E-02
Rhobtb1	69288	-0.06	0.07	4.38E-01	-0.20	0.09	8	8.58E-01	4.23	2.35E-05	1.69E-02
Zdhhc19	245308	-0.39	0.31	1.98E-01	-0.99	0.20	7	7.05E-01	-4.16	3.13E-05	1.99E-02
Slc38a1	105727	0.05	0.03	1.13E-01	-0.01	0.11	8	5.96E-01	4.16	3.15E-05	1.99E-02
Sh2d5	230863	-0.03	0.06	5.88E-01	-0.15	0.09	7	9.13E-01	4.15	3.26E-05	1.99E-02
Nfil3	18030	0.09	0.12	4.52E-01	-0.15	0.33	8	8.62E-01	4.12	3.80E-05	2.12E-02
Phlda3	27280	0.15	0.14	2.87E-01	-0.13	0.43	8	7.79E-01	4.12	3.81E-05	2.12E-02
Ruvbl1	56505	0.00	0.04	9.96E-01	-0.08	0.08	8	1.00E+00	-4.08	4.51E-05	2.31E-02
Pitpnm2	19679	-0.04	0.06	5.48E-01	-0.16	0.08	8	9.04E-01	4.08	4.54E-05	2.31E-02
Cx3cr1	13051	0.00	0.08	9.81E-01	-0.16	0.16	8	9.98E-01	-4.05	5.23E-05	2.56E-02
Srrt	83701	0.01	0.07	9.01E-01	-0.13	0.15	7	9.86E-01	4.01	5.96E-05	2.59E-02
Csdc2	105859	0.13	0.10	1.68E-01	-0.06	0.32	8	6.75E-01	4.01	6.11E-05	2.59E-02
Adgrb2	230775	0.02	0.05	6.84E-01	-0.08	0.13	8	9.39E-01	4.01	6.17E-05	2.59E-02
Gadd45g	23882	-0.12	0.08	1.16E-01	-0.28	0.03	8	6.02E-01	-4.00	6.25E-05	2.59E-02
Tm4sf1	17112	0.00	0.09	9.81E-01	-0.18	0.17	8	9.98E-01	4.00	6.37E-05	2.59E-02
Sertad1	55942	-0.26	0.17	1.18E-01	-0.59	0.07	8	6.03E-01	-3.97	7.27E-05	2.84E-02
Pkhd1l1	192190	0.35	0.24	1.38E-01	-0.11	0.81	8	6.34E-01	3.95	7.66E-05	2.84E-02
Cdhr1	170677	0.29	0.26	2.59E-01	-0.21	0.80	8	7.60E-01	3.95	7.68E-05	2.84E-02
Zswim5	74464	-0.04	0.07	5.75E-01	-0.17	0.09	7	9.12E-01	-3.94	7.99E-05	2.87E-02
Ece1	230857	-0.03	0.06	6.24E-01	-0.15	0.09	7	9.21E-01	3.91	9.25E-05	3.23E-02
Rgs2	19735	-0.06	0.05	1.78E-01	-0.16	0.03	8	6.86E-01	-3.90	9.55E-05	3.24E-02
Dock3	208869	-0.08	0.07	2.64E-01	-0.22	0.06	8	7.64E-01	-3.82	1.32E-04	4.37E-02
Tcn2	21452	0.05	0.03	1.13E-01	-0.01	0.10	8	5.96E-01	-3.81	1.39E-04	4.47E-02
Mycbpap	104601	0.42	0.22	5.64E-02	-0.01	0.85	8	4.80E-01	3.79	1.50E-04	4.71E-02

Table S7. Exploratory meta-analysis results: The full results from the exploratory meta-analysis of antipsychotic effects on the frontal cortex which used data from both rodents and macaques (13,121 genes (10,617 of which had macaque data), 13,107 stable meta-analysis estimates). *This .xlsx file includes two worksheets: 1) The worksheet "ColumnDefinitions" provides the definitions for the variables present in each column in "Exploratory_RodentMacaque_MetaAnalysis". 2) The worksheet "Exploratory_RodentMacaque_MetaAnalysis" provides the full meta-analysis results, with each row*

representing the results for one gene, and each column providing either gene annotation or meta-analysis statistical output. The results are ordered by p-value, so that the top rows in the worksheet are the genes with the smallest p-values.

Table S8. Exploratory meta-analysis results: All 70 genes consistently upregulated in the frontal cortex following antipsychotic treatment in an exploratory meta-analysis including rodents and macaques (FDR<0.05). *Table follows the conventions of* ***Table S3****. Results are ordered by estimated Log2FC (Log2 Fold Change of Antipsychotic treatment vs. Control); a higher Log2FC indicates upregulation of the gene within the frontal cortex following antipsychotic treatment compared to the control group. Given gene symbols are the mouse symbol for the respective gene. Full results for all genes can be found in* ***Table S****7.*

Gene Symbol	Mouse Entrez Gene ID	Log2FC estimate	SE	pval	CI_lb	CI_ub	# Comparisons	FDR
Wfikkn1	215001	0.29	0.08	4.37E-04	0.13	0.45	10	4.90E-02
Gjb1	14618	0.27	0.06	4.04E-06	0.16	0.39	8	4.41E-03
Nkx6-2	14912	0.24	0.06	1.61E-05	0.13	0.36	11	8.76E-03
Mfap2	17150	0.24	0.04	2.09E-10	0.16	0.31	11	2.74E-06
Itgal	16408	0.23	0.05	2.17E-06	0.13	0.32	10	2.84E-03
Igfbp5	16011	0.22	0.05	2.27E-05	0.12	0.32	11	1.03E-02
Isg15	100038882	0.21	0.06	4.07E-04	0.10	0.33	11	4.73E-02
Prok1	246691	0.21	0.05	7.66E-05	0.10	0.31	7	1.90E-02
Calb2	12308	0.20	0.04	9.25E-07	0.12	0.28	11	1.65E-03
Mroh7	381538	0.20	0.05	1.15E-04	0.10	0.30	9	2.33E-02
Il13ra1	16164	0.19	0.04	5.93E-06	0.11	0.28	7	4.86E-03
Gh	14599	0.19	0.05	1.11E-04	0.09	0.29	8	2.30E-02
Slc18a3	20508	0.18	0.05	6.85E-05	0.09	0.27	8	1.80E-02
Tdo2	56720	0.18	0.04	7.78E-06	0.10	0.26	8	6.01E-03
Adora2b	11541	0.17	0.04	4.73E-06	0.10	0.25	11	4.58E-03
1700010I14Rik	66931	0.17	0.04	3.10E-05	0.09	0.25	8	1.16E-02
Osgin1	71839	0.17	0.04	2.25E-05	0.09	0.25	11	1.03E-02
Gpr84	80910	0.17	0.04	4.27E-05	0.09	0.25	8	1.27E-02
Slc14a1	108052	0.17	0.04	4.88E-06	0.10	0.24	11	4.58E-03
Ccdc71	72454	0.17	0.03	9.08E-07	0.10	0.23	10	1.65E-03
Camk2n2	73047	0.17	0.04	1.68E-04	0.08	0.25	11	2.92E-02
Wscd1	216881	0.16	0.04	2.65E-05	0.09	0.23	11	1.12E-02

Slc39a2	*214922*	*0.16*	*0.05*	*4.06E-04*	*0.07*	*0.25*	*8*	*4.73E-02*
Slc1a5	*20514*	*0.16*	*0.04*	*1.95E-05*	*0.09*	*0.23*	*8*	*9.83E-03*
Serinc5	*218442*	*0.16*	*0.03*	*2.98E-07*	*0.10*	*0.22*	*11*	*9.77E-04*
Sfrp5	*54612*	*0.15*	*0.04*	*3.83E-04*	*0.07*	*0.24*	*11*	*4.57E-02*
Chad	*12643*	*0.15*	*0.04*	*1.05E-04*	*0.08*	*0.23*	*10*	*2.26E-02*
F10	*14058*	*0.15*	*0.04*	*2.17E-05*	*0.08*	*0.22*	*10*	*1.03E-02*
Parp12	*243771*	*0.15*	*0.04*	*2.80E-04*	*0.07*	*0.23*	*11*	*3.89E-02*
Spc24	*67629*	*0.15*	*0.04*	*9.00E-05*	*0.07*	*0.22*	*10*	*2.11E-02*
Aspa	*11484*	*0.14*	*0.04*	*2.15E-04*	*0.07*	*0.22*	*11*	*3.21E-02*
Hk2	*15277*	*0.14*	*0.04*	*3.21E-04*	*0.07*	*0.22*	*11*	*4.13E-02*
Megf6	*230971*	*0.14*	*0.04*	*1.15E-04*	*0.07*	*0.22*	*11*	*2.33E-02*
Gpr137b	*83924*	*0.14*	*0.04*	*7.33E-05*	*0.07*	*0.21*	*9*	*1.85E-02*
Gtse1	*29870*	*0.14*	*0.04*	*1.08E-04*	*0.07*	*0.21*	*8*	*2.29E-02*
Mrgprf	*211577*	*0.14*	*0.03*	*4.05E-05*	*0.07*	*0.21*	*11*	*1.27E-02*
Scarf2	*224024*	*0.14*	*0.04*	*2.66E-04*	*0.07*	*0.22*	*11*	*3.79E-02*
Nkain3	*269513*	*0.13*	*0.03*	*6.34E-05*	*0.07*	*0.20*	*9*	*1.73E-02*
Slc29a3	*71279*	*0.13*	*0.03*	*4.15E-05*	*0.07*	*0.20*	*11*	*1.27E-02*
Stx2	*13852*	*0.13*	*0.04*	*2.13E-04*	*0.06*	*0.20*	*11*	*3.21E-02*
Arhgap45	*70719*	*0.13*	*0.03*	*1.78E-04*	*0.06*	*0.20*	*11*	*2.92E-02*
Polr1g	*70333*	*0.13*	*0.03*	*1.99E-04*	*0.06*	*0.19*	*7*	*3.11E-02*
Arhgef10	*234094*	*0.12*	*0.03*	*1.02E-04*	*0.06*	*0.19*	*10*	*2.24E-02*
Rbp7	*63954*	*0.12*	*0.03*	*1.82E-04*	*0.06*	*0.19*	*7*	*2.92E-02*
Nde1	*67203*	*0.12*	*0.03*	*9.34E-05*	*0.06*	*0.18*	*11*	*2.13E-02*
Kit	*16590*	*0.12*	*0.03*	*1.00E-05*	*0.06*	*0.17*	*11*	*6.93E-03*
Cdh3	*12560*	*0.12*	*0.03*	*3.56E-04*	*0.05*	*0.18*	*11*	*4.36E-02*
Fam234a	*106581*	*0.12*	*0.03*	*1.56E-04*	*0.06*	*0.18*	*8*	*2.80E-02*
Cmtm3	*68119*	*0.11*	*0.03*	*2.15E-04*	*0.05*	*0.17*	*11*	*3.21E-02*
Psme2	*19188*	*0.11*	*0.02*	*1.71E-06*	*0.07*	*0.16*	*9*	*2.50E-03*
Cd180	*17079*	*0.11*	*0.03*	*4.19E-05*	*0.06*	*0.16*	*11*	*1.27E-02*
Hnrnpl	*15388*	*0.11*	*0.03*	*1.69E-04*	*0.05*	*0.17*	*8*	*2.92E-02*
Fgfr1	*14182*	*0.11*	*0.03*	*4.08E-05*	*0.05*	*0.16*	*11*	*1.27E-02*
Nrros	*224109*	*0.10*	*0.03*	*9.60E-05*	*0.05*	*0.15*	*11*	*2.14E-02*

Klhl13	67455	0.10	0.03	3.87E-04	0.04	0.16	10	4.57E-02
Atad2	70472	0.10	0.03	1.77E-04	0.05	0.15	11	2.92E-02
Tmem134	66990	0.10	0.03	3.06E-04	0.04	0.15	11	4.06E-02
Lsm4	50783	0.09	0.02	1.51E-04	0.05	0.14	11	2.79E-02
Nme3	79059	0.09	0.03	3.65E-04	0.04	0.14	11	4.44E-02
Rin2	74030	0.09	0.02	2.39E-04	0.04	0.14	11	3.52E-02
Ctdp1	67655	0.09	0.02	9.40E-05	0.04	0.13	11	2.13E-02
Slc22a5	20520	0.09	0.02	3.16E-04	0.04	0.13	11	4.13E-02
Mgat3	17309	0.08	0.02	1.25E-05	0.05	0.12	11	7.45E-03
Hnrnpa1	15382	0.08	0.02	3.73E-04	0.04	0.13	10	4.49E-02
Maf1	68877	0.08	0.02	8.66E-05	0.04	0.12	11	2.08E-02
Rpl27a	26451	0.08	0.02	6.98E-05	0.04	0.11	8	1.80E-02
Acadvl	11370	0.07	0.02	1.38E-04	0.04	0.11	11	2.62E-02
Lypla2	26394	0.07	0.02	6.70E-05	0.04	0.11	11	1.79E-02
Ciao1	26371	0.07	0.02	2.10E-04	0.03	0.11	11	3.21E-02
Dnajc11	230935	0.06	0.02	2.97E-04	0.03	0.09	11	4.03E-02

Table S9. Planned meta-analysis results: All 47 genes consistently downregulated in the frontal cortex following antipsychotic treatment in an exploratory meta-analysis including rodents and macaques (FDR<0.05). *Table follows the conventions of* ***Table S3****. Results are ordered by estimated Log2FC (Log2 Fold Change of Antipsychotic treatment vs. Control); a lower Log2FC indicates downregulation of the gene within the frontal cortex following antipsychotic treatment compared to the control group. Full results for all genes can be found in* ***Table S****7.*

Gene Symbol	Mouse Entrez Gene ID	Log2FC estimate	SE	pval	CI_lb	CI_ub	# Comparisons	FDR
Dmkn	73712	-0.80	0.15	1.98E-07	-1.10	-0.50	7	8.66E-04
Ms4a8a	64381	-0.25	0.04	6.28E-09	-0.33	-0.16	7	4.12E-05
Dglucy	217830	-0.21	0.06	3.39E-04	-0.33	-0.10	7	4.23E-02
Nr0b1	11614	-0.17	0.05	4.23E-04	-0.27	-0.08	7	4.78E-02
Fabp4	11770	-0.17	0.04	5.36E-05	-0.25	-0.09	7	1.50E-02
Cth	107869	-0.16	0.04	1.67E-04	-0.24	-0.07	11	2.92E-02

Or51e2	*170639*	*-0.15*	*0.04*	*3.24E-04*	*-0.24*	*-0.07*	*8*	*4.13E-02*
Pus7l	*78895*	*-0.15*	*0.04*	*1.67E-05*	*-0.22*	*-0.08*	*10*	*8.76E-03*
Proser3	*333193*	*-0.15*	*0.04*	*2.64E-04*	*-0.23*	*-0.07*	*9*	*3.79E-02*
Cdhr5	*72040*	*-0.15*	*0.04*	*4.21E-04*	*-0.23*	*-0.07*	*7*	*4.78E-02*
Opn3	*13603*	*-0.15*	*0.04*	*3.76E-05*	*-0.22*	*-0.08*	*11*	*1.27E-02*
Trib1	*211770*	*-0.14*	*0.04*	*1.82E-04*	*-0.22*	*-0.07*	*11*	*2.92E-02*
Kcnh5	*238271*	*-0.14*	*0.04*	*1.22E-04*	*-0.21*	*-0.07*	*11*	*2.42E-02*
Tatdn1	*69694*	*-0.14*	*0.03*	*5.34E-06*	*-0.19*	*-0.08*	*10*	*4.67E-03*
Ar	*11835*	*-0.13*	*0.03*	*1.24E-04*	*-0.19*	*-0.06*	*11*	*2.43E-02*
Tmem254	*66039*	*-0.12*	*0.03*	*1.45E-04*	*-0.19*	*-0.06*	*7*	*2.71E-02*
Susd2	*71733*	*-0.12*	*0.03*	*2.77E-04*	*-0.18*	*-0.06*	*8*	*3.89E-02*
Ncr1	*17086*	*-0.12*	*0.03*	*3.44E-05*	*-0.18*	*-0.06*	*7*	*1.25E-02*
Slc35g2	*245020*	*-0.11*	*0.02*	*3.46E-06*	*-0.16*	*-0.06*	*9*	*4.13E-03*
Snx16	*74718*	*-0.11*	*0.03*	*1.73E-04*	*-0.16*	*-0.05*	*11*	*2.92E-02*
Chic1	*12212*	*-0.11*	*0.02*	*1.01E-06*	*-0.15*	*-0.06*	*10*	*1.65E-03*
Nudt7	*67528*	*-0.11*	*0.03*	*4.17E-04*	*-0.16*	*-0.05*	*11*	*4.78E-02*
Ttc39b	*69863*	*-0.10*	*0.02*	*4.51E-05*	*-0.15*	*-0.05*	*11*	*1.32E-02*
Wdpcp	*216560*	*-0.10*	*0.02*	*2.88E-05*	*-0.14*	*-0.05*	*11*	*1.14E-02*
Mrps30	*59054*	*-0.10*	*0.02*	*5.91E-07*	*-0.14*	*-0.06*	*11*	*1.55E-03*
Lrrc8c	*100604*	*-0.10*	*0.02*	*2.97E-05*	*-0.14*	*-0.05*	*11*	*1.15E-02*
Polr3c	*74414*	*-0.10*	*0.03*	*3.36E-04*	*-0.15*	*-0.04*	*11*	*4.23E-02*
Errfi1	*74155*	*-0.09*	*0.02*	*1.35E-05*	*-0.14*	*-0.05*	*11*	*7.68E-03*
Sdhaf3	*71238*	*-0.09*	*0.03*	*2.82E-04*	*-0.14*	*-0.04*	*11*	*3.89E-02*
Fam13c	*71721*	*-0.09*	*0.02*	*9.57E-06*	*-0.13*	*-0.05*	*10*	*6.93E-03*
Syt4	*20983*	*-0.09*	*0.02*	*5.14E-05*	*-0.14*	*-0.05*	*11*	*1.47E-02*
Cwc22	*80744*	*-0.09*	*0.02*	*2.75E-05*	*-0.13*	*-0.05*	*10*	*1.13E-02*
Osbpl8	*237542*	*-0.09*	*0.02*	*2.39E-05*	*-0.13*	*-0.05*	*11*	*1.04E-02*
Zdhhc5	*228136*	*-0.09*	*0.02*	*8.70E-05*	*-0.13*	*-0.04*	*11*	*2.08E-02*
Psmd12	*66997*	*-0.09*	*0.02*	*1.19E-05*	*-0.13*	*-0.05*	*11*	*7.45E-03*
Tasp1	*75812*	*-0.08*	*0.02*	*1.79E-04*	*-0.13*	*-0.04*	*11*	*2.92E-02*

Gtf3c3	*98488*	*-0.08*	*0.02*	*2.45E-04*	*-0.13*	*-0.04*	*10*	*3.57E-02*
Phf14	*75725*	*-0.08*	*0.02*	*2.98E-04*	*-0.13*	*-0.04*	*11*	*4.03E-02*
Prickle1	*106042*	*-0.08*	*0.02*	*3.18E-04*	*-0.12*	*-0.04*	*11*	*4.13E-02*
Pwp2	*110816*	*-0.08*	*0.02*	*1.56E-04*	*-0.12*	*-0.04*	*11*	*2.80E-02*
Rbm12	*75710*	*-0.08*	*0.02*	*1.31E-04*	*-0.12*	*-0.04*	*7*	*2.52E-02*
Hace1	*209462*	*-0.08*	*0.02*	*3.01E-04*	*-0.12*	*-0.03*	*11*	*4.03E-02*
Tfb2m	*15278*	*-0.07*	*0.02*	*1.96E-04*	*-0.11*	*-0.03*	*11*	*3.10E-02*
Dctn4	*67665*	*-0.07*	*0.02*	*4.20E-05*	*-0.11*	*-0.04*	*10*	*1.27E-02*
Aptx	*66408*	*-0.07*	*0.02*	*4.00E-05*	*-0.11*	*-0.04*	*11*	*1.27E-02*
Camsap2	*67886*	*-0.07*	*0.02*	*1.23E-05*	*-0.10*	*-0.04*	*11*	*7.45E-03*
Ipo7	*233726*	*-0.06*	*0.02*	*3.48E-04*	*-0.09*	*-0.03*	*11*	*4.30E-02*

Table S10: Planned Rodent Meta-Analysis: Non-directional fast Gene Set Enrichment Analysis (fGSEA) results (10,147 gene sets). *This .xlsx file includes two worksheets: 1) The worksheet "ColumnDefinitions" provides the definitions for the variables present in each column in "RodentMetaAnalysis_nondirectional_GSEA". 2) The worksheet "RodentMetaAnalysis_nondirectional_GSEA" provides the full fGSEA results, with each row representing the results for one gene set, and each column providing the fGSEA statistical output. The results are ordered by p-value, so that the top rows in the worksheet are the gene sets with the smallest p-values.*

Table S11: Planned Rodent Meta-Analysis: Directional fast Gene Set Enrichment Analysis (fGSEA) results (10,147 gene sets). *This .xlsx file includes two worksheets: 1) The worksheet "ColumnDefinitions" provides the definitions for the variables present in each column in "RodentMetaAnalysis_DirectionalGSEA". 2) The worksheet "RodentMetaAnalysis_DirectionalGSEA" provides the full fGSEA results, with each row representing the results for one gene set, and each column providing the fGSEA statistical output. The results are ordered by p-value, so that the top rows in the worksheet are the gene sets with the smallest p-values.*

Table S12: Exploratory Rodent and Macaque Meta-Analysis: Non-directional fast Gene Set Enrichment Analysis (fGSEA) results (10,417 gene sets). *This .xlsx file includes two worksheets: 1) The worksheet "Column Definitions" provides the definitions for the variables present in each column in "Exploratory_RodentMacaque_GSEA_NonDirectional". 2) The worksheet "Exploratory_RodentMacaque_GSEA_NonDirectional" provides the full fGSEA results, with each row representing the results for one gene set, and each column providing the fGSEA statistical output. The results are ordered by p-value, so that the top rows in the worksheet are the gene sets with the smallest p-values.*

Table S13: Exploratory Rodent and Macaque Meta-Analysis: Directional fast Gene Set Enrichment Analysis (fGSEA) results (10,417 gene sets). *This .xlsx file includes two worksheets: 1) The worksheet "Column Definitions" provides the definitions for the variables present in each column in "Exploratory_RodentMacaque_GSEA_Directional". 2) The worksheet "Exploratory_RodentMacaque_GSEA_Directional" provides the full fGSEA results, with each row representing the results for one gene set, and each column providing the fGSEA statistical output. The results are ordered by p-value, so that the top rows in the worksheet are the gene sets with the smallest p-values.*

Supplemental Figures

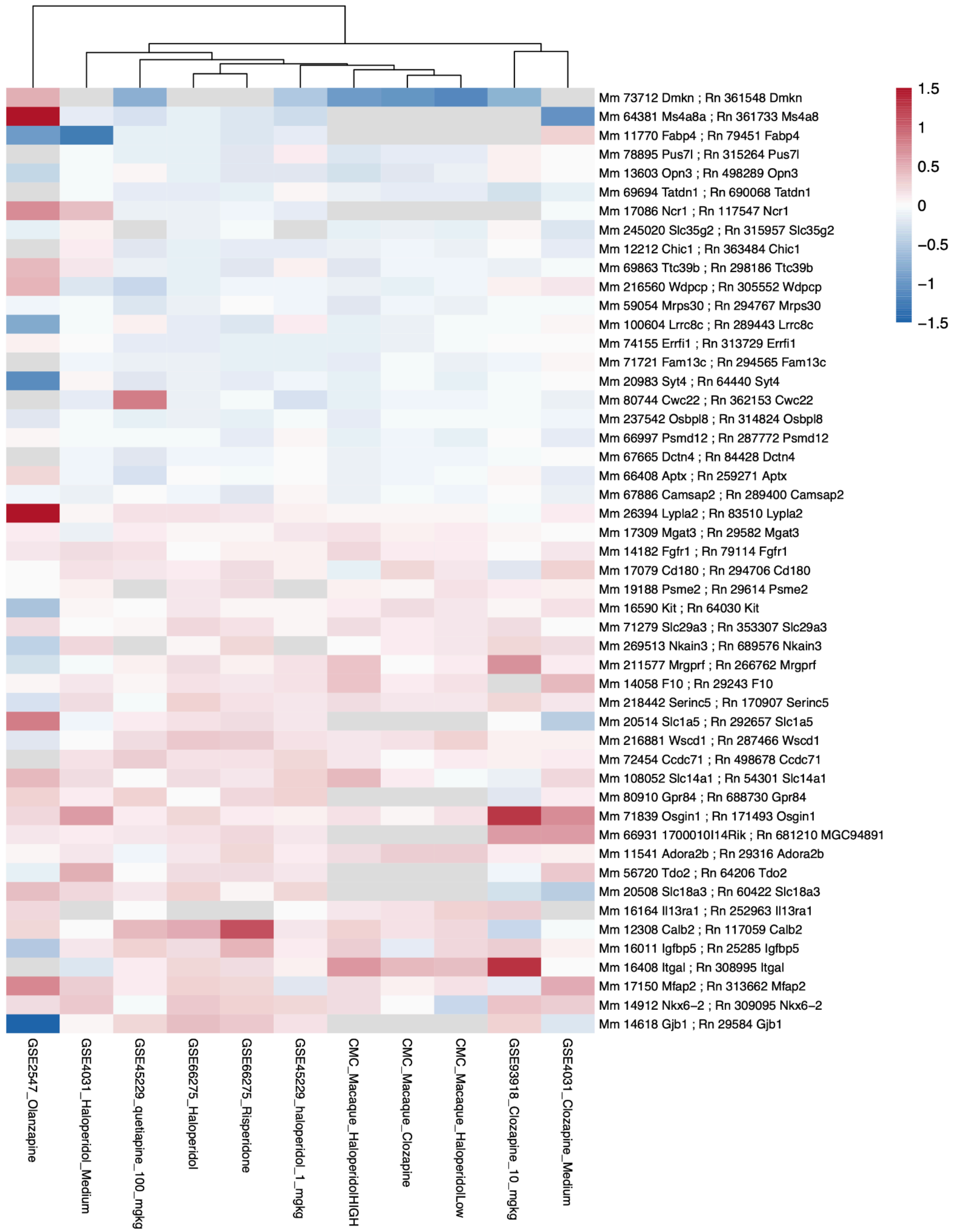

Figure S1. A hierarchically-clustered heatmap illustrating the antipsychotic vs. control effect sizes across all group comparisons for each of the top 50 differentially expressed genes (DEGs) in the exploratory meta-analysis using both rodent and macaque data. *The heatmap displays the antipsychotic vs. control effect sizes (Log(2) fold changes or Log2FCs) for each of the 50 differentially expressed genes identified through the meta-analysis as significantly differentially expressed (FDR<0.05). Results are scaled and color-coded by log2FC, ranging from -1.5 to +1.5 (blue to red; downregulation to upregulation). Rows represent individual genes, annotated with the ortholog mouse (Mm) and rat (Rn) Entrez ids and gene symbols. Each column corresponds to a specific treatment vs. control comparison, identified by Gene Expression Omnibus dataset id (GSE…) and treatment used. Hierarchical clustering is applied and used to group columns with similar expression profiles.*
